# Changes in soft tissue dimensions following horizontal guided bone regeneration with a split-thickness flap design – evaluation of 8 cases with a digital method

**DOI:** 10.1186/s13005-024-00456-8

**Published:** 2024-09-28

**Authors:** Kristof Somodi, Andrea Dobos, Ferenc Bartha, Eleonora Solyom, Peter Windisch, Daniel Palkovics, Balint Molnar

**Affiliations:** Department of Periodontology, Semmelweis University, Szentkirályi Street 47, Budapest, 1088 Hungary

**Keywords:** Soft tissue alterations, Horizontal ridge augmentation, Split-thickness flap, Intraoral scan, CBCT analysis

## Abstract

**Background:**

Peri-implant soft tissue corrections are often indicated following alveolar ridge augmentation, due to the distortion of the keratinized mucosa at the area of augmentation. The objective of the current study was to evaluate the dimensional soft tissue changes following horizontal guided bone regeneration (GBR) utilizing 3D digital data.

**Methods:**

8 mandibular surgical sites with horizontal alveolar ridge deficiencies were treated utilizing a resorbable collagen membrane and a split-thickness flap design. Baseline and 6-month follow-up cone-beam computed tomography (CBCT) scans were reconstructed as 3D virtual models and were superimposed with the corresponding intraoral scan. Linear changes of supracrestal vertical- horizontal soft tissue alterations were measured in relation to the alveolar crest at the mesial- middle- and distal aspect of the surgical area. Soft tissue dimensions were measured at baseline and at 6-month follow-up.

**Results:**

Preoperative supracrestal soft tissue height measured midcrestally averaged at 2.37 mm ± 0.68 mm, 2.37 mm ± 0.71 mm and 2.64 mm ± 0.87 mm at the mesial-, middle- and distal planes. Whereas postoperative supracrestal soft tissue height was measured at 2.62 mm ± 0.72 mm, 2.67 mm ± 0.67 mm and 3.69 mm ± 1.02 mm at the mesial, middle and distal planes, respectively. Supracrestal soft tissue width changed from 2.14 mm ± 0.72 mm to 2.47 mm ± 0.46 mm at the mesial, from 1.72 mm ± 0.44 mm to 2.07 mm ± 0.67 mm and from 2.15 mm ± 0.36 mm to 2.36 mm ± 0.59 mm at the mesial, middle and distal planes, respectively. Additionally the buccal horizontal displacement of supracrestal soft tissues could be observed.

**Conclusions:**

The current study did not report significant supracrestal soft tissue reduction following horizontal GBR with a split-thickness flap. Even though there was a slight increase in both vertical and horizontal dimensions, differences are clinically negligible.

**Trail registration:**

The trail was approved by the U.S. National Library of Medicine (www.clinicaltrials.gov); trial registration number: NCT05538715; registration date: 09/09/2022.

## Background

Over the course of the past 20 years guided bone regeneration (GBR) has become well-established method for reconstructing lost alveolar structures [4, 24]. Literature data has also shown that long-term success of implants placed into augmented sites does not differ significantly to those placed into native bone [7, 13]. Even though various aspects of GBR are well researched, there is less information available regarding soft tissue changes following ridge augmentation procedures. In the clinical practice reduction of keratinized tissue width- and thickness is a well-known occurrence, consequently, soft tissue augmentation and re-establishment of keratinized mucosa width following alveolar ridge reconstructions is often required. Yet, there is only limited literature data available reporting on soft tissue alterations and the factors that influence morphological changes in keratinized tissues after ridge augmentation procedures. It can be hypothesized that the reduction of the keratinized mucosa and distortion of the vestibule following ridge augmentation procedures occur due to extensive flap mobilization. In order to avoid severe vestibular distortion, Windisch et al. [29, 30] have suggested the application of a split-thickness flap design - instead of a conventional full-thickness flap – to be used in conjunction with GBR.

The lack of adequate peri-implant soft tissue dimensions – i.e., the supracrestal soft tissue thickness and the width of peri-implant keratinized mucosa (PIKM) – results in reduced long-term implant success rates [6, 31]. As early as 1996 [2], it was shown in a preclinical study that the reduction of supracrestal soft tissue thickness below 2 mm led to a marginal peri-implant bone loss. In 2015, Linkevicius et al. [14] showed that thick (> 2 mm) supracrestal soft tissue dimensions resulted in significantly less marginal bone loss. While a narrow band of PIKM facilitates plaque accumulation, increasing the rate of peri-implant mucositis and eventually peri-implantitis [3, 18, 20, 27, 28]. To avoid peri-implant soft tissue related complications, the surgical reconstruction of the keratinized mucosa at the edentulous ridge after augmentation is often necessary [5, 12, 19, 23, 25].

Different methods can be found in the literature for the measurement of supracrestal soft tissues. Width of the supracrestal soft tissues can be easily assess clinically with the use of periodontal probes or calipers. Supracrestal soft tissue thickness on the other hand is most commonly measured by the means of transmucosal probing (bone sounding) which is a relatively invasive method [11]. Alternatively, ultrasonographic devices or the superimposition of cone-beam computed tomography (CBCT) scans and intraoral scans (IOSs) can be utilized to assess supracrestal soft tissue thickness in a non-invasive way [11, 22]. A previous study by Di Raimondo et al. [9], analyzed soft tissue changes occurring after simultaneous horizontal GBR and implant placement utilizing digitalized casts. After superimposition of baseline, 4-month and 12-month casts authors performed horizontal cross sectional linear measurements. Authors have concluded that soft tissue contours have increased after horizontal GBR. However, measurements represent the cumulative change in alveolar ridge dimensions (hard tissue and soft tissue changes) rather than solely analyzing soft tissue alterations.

In a previous study, hard tissue changes following horizontal GBR have been investigated utilizing a 3D methodology [16]. However, concomitant soft tissue alterations were not analyzed. With the combination of 3D reconstructed CBCT scans and IOSs, digital hybrid models can be generated [17]. Digital hybrid models depict all relevant anatomical structures –i.e., teeth, alveolar bone, soft tissues – separately, allowing to analyze hard and soft tissue changes independently from one another.

Hence the aim of our study was to evaluate soft tissue dimensional changes following horizontal GBR utilizing 3D digital hybrid models.

## Methods

### Study design

This prospective single-center case series included a total of 8 surgical sites in the posterior mandible. Data included in the current paper are derived from a single group of a larger ongoing randomized clinical trial. The current pilot study investigated the supracrestal soft tissue alterations following horizontal GBR, before second stage dental implant placement. The study followed the PROCESS guidelines checklist (originally published in 2016, revised in 2018) [1]. The study protocol was approved by the Semmelweis University Regional and Institutional Committee of Science and Research Ethics (Approval Number: SE RKEB 145/2018) and the U.S. National Library of Medicine (www.clinicaltrials.gov; trial registration number: NCT05538715; registration date: 09/09/2022). The study was conducted in full accordance with the Declaration of Helsinki of 1975, revised in 2013 [10]. Surgical interventions were performed with the understanding and written informed consent of every participant.

### Patient selection

Participants enrolled in the study, were treated at the Department of Periodontology, Semmelweis University. In the included cases, horizontal ridge augmentation in the posterior mandible was necessary for a prosthetically driven implant placement. Baseline defect morphologies were classified according to the HVC (horizontal, vertical, combined) ridge deficiency classification [26].

Exclusion criteria were: (i) presence of general medical conditions contraindicating surgical treatment; (ii) age < 20 years, (iii) smoking; (iv) untreated periodontitis with high levels of residual inflammation (full mouth bleeding score > 25%); (v) inadequate oral hygiene (full mouth plaque score > 25%) and (iv) vertical or combined alveolar ridge deficiencies.

CBCT images were taken with a Planmeca ProMax 3D Plus and a Planmeca Viso G7 device (Planmeca Oy, Helsinki, Finland) (FOV: 10 × 10 cm, voxel size: 150 μm) prior to and 6 months following the augmentation procedure. Intraoral scans were acquired with Planmeca Emerald S (Planmeca Oy, Helsinki, Finland) at baseline and 6-month follow-up.

### Surgical procedure

Steps of the surgical protocol have been described in detail elsewhere [16]. Briefly, first a mid-crestal incision was made, thereafter a double layer (mucosa and periosteum), split-thickness flap was raised on the buccal aspect. While on the lingual aspect a full-thickness mucoperiosteal flap was raised. To maintain the blood supply of the periosteum, vertical releasing incisions were avoided. Autogenous bone chips were harvested by a single use bone collector device (Safescraper Twist, Meta, Reggio Emilia, Italy) locally without the preparation of a second surgical site and were mixed with a bovine-derived xenograft (Bio_Oss, Gesitlich, Wolhusen, Germany) in a 1:1 ratio. A resorbable collagen membrane (BioGide, Geistlich, Wolhusan, Germany) was shaped and fixated on the lingual aspect with titanium micro-screws (Pro-fix, Osteogenics, Lubbock, USA). Thereafter, the composite graft was compacted on the residual ridge and the collagen membrane was folded over and subsequently fixed with titanium pins (Ustomed, Tuttlingen, Germany).

**Fig. 1:**
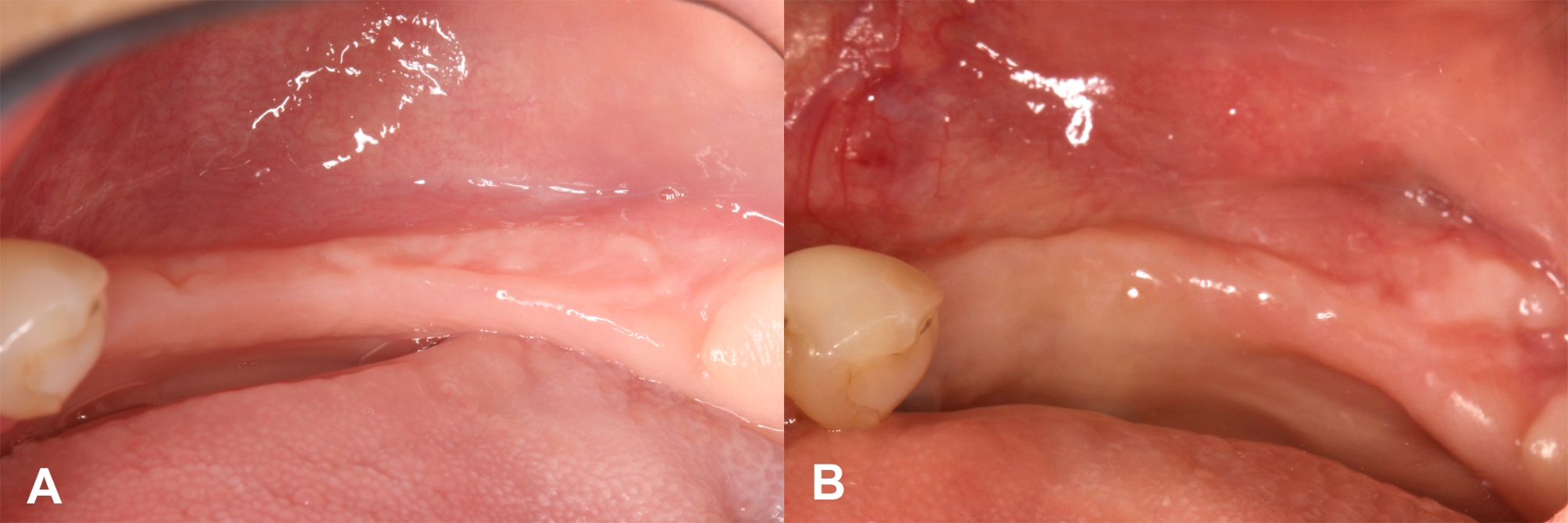
Clinical situation of the supracrestal keratinized mucosa before and after hard tissue augmentation. (**A**) Baseline. (**B**) 6-month follow-up

Double-layer wound closure was carried out, first the buccal periosteal layer was sutured to the lingual flap with a 3–0 expanded polytetrafluoroethylene (e-PTFE) suturing material (Cytoplast, Osteogenics, Lubbock, USA). The buccal mucosal layer was also sutured to the lingual flap with horizontal mattress sutures and single interrupted sutures using a 4–0 non-resorbable e-PTFE suturing material (Cytoplast, Osteogenics, Lubbock, USA). Baseline and 6-month follow-up of keratinized soft tissue situations are visible in Fig. 1.

**Table 1:** Preoperative and postoperative vertical soft tissue dimensions at the mesial, middle, and distal planes of the augmented area

Patient	Vertical preop mesial (mm)	Vertical postop mesial (mm)	Vertical preop mid (mm)	Vertical postop mid (mm)	Vertical preop distal (mm)	Vertical postop distal (mm)
1	3,509	3,733	3,702	3,551	4,106	5,501
2	3,194	3,199	2,804	3,458	3,786	4,806
3	1,7	2,048	1,238	2,133	2,111	3,916
4	2,014	2,21	1,873	2,334	2,565	3,155
5	2,61	1,929	2,186	3,271	2,451	3,375
6	1,811	1,998	2,282	2,576	2,517	3,159
7	1,855	2,418	2,308	2,281	1,629	2,346
8	2,301	3,434	2,563	1,738	1,924	3,249
Mean ± Standard deviation	2.37 ± 0.68	2.62 ± 0.72	2.37 ± 0.71	2.67 ± 0.67	2.64 ± 0.87	3.69 ± 1.02

### Digital data processing

Baseline and 6-month follow-up CBCT scans were segmented in an open-source radiographic image processing software (3D Slicer, www.slicer.org) using a dedicated semi-automatic image segmentation method [15]. Anatomical structures (teeth, alveolar bone and nerves) were segmented separately, constituting individual components of the model. The output of image segmentation is a 3D virtual model of the dento-alveolar hard tissues.

**Fig. 2:**
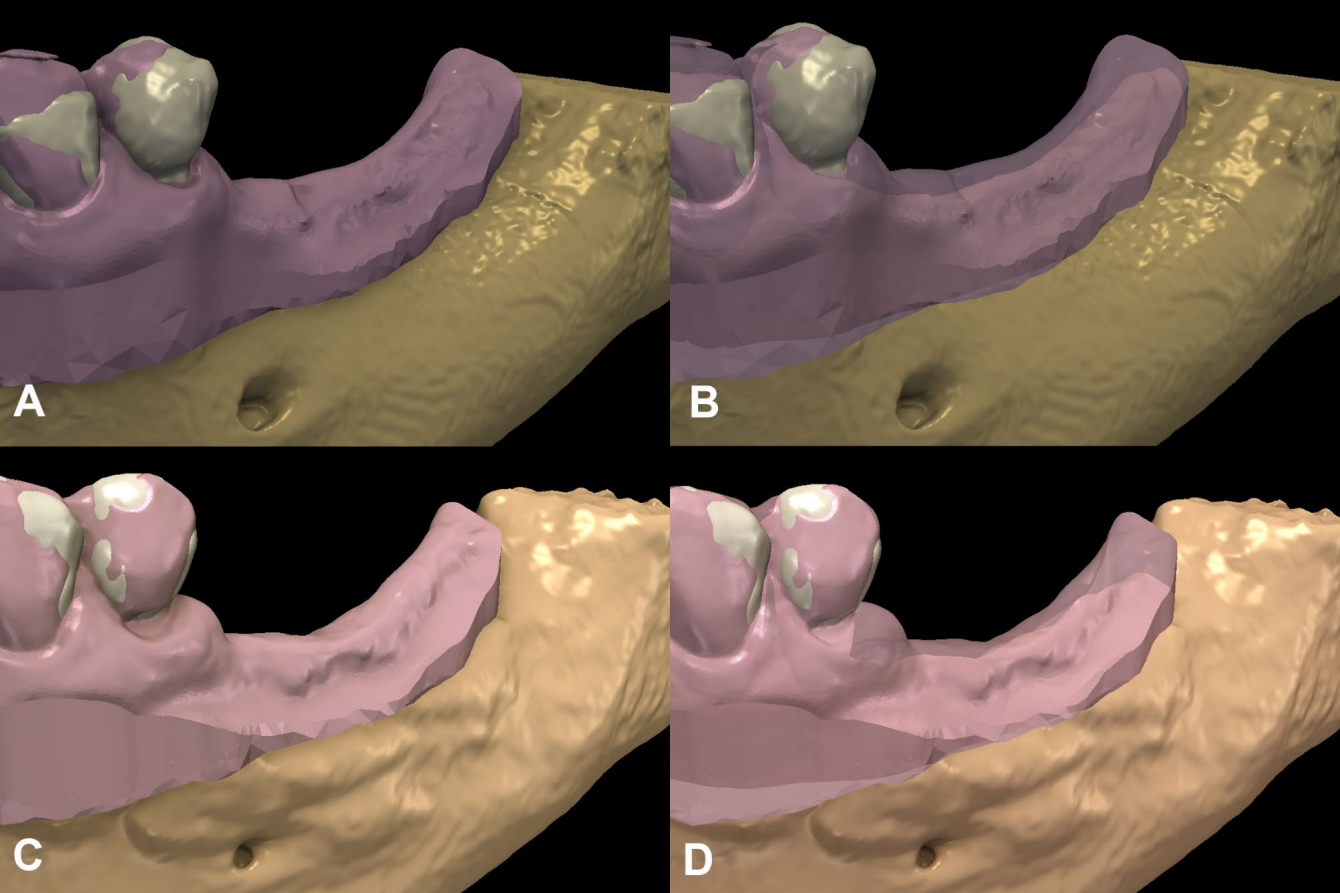
Three-dimensional digital hybrib models. (**A-B**) Virtual depiction of the baseline situation. (**C-D**) Virtual depiction of the 6-month follow-up situation

Following segmentation, standard tessellation language (.stl) files of IOSs were superimposed with segmented 3D models, using identical landmark registration. Corresponding mark-up points were placed on fixed anatomical landmarks (cusps or incisor edges of teeth) (Fig. 2). Alignment of the two models were inspected by two individual investigators.

**Table 2:** Preoperative and postoperative horizontal soft tissue dimensions at the mesial, the middle, and distal parts of the augmented area

Patient	Horizontal preop mesial (mm)	Horizontal postop mesial (mm)	Horizontal preop mid (mm)	Horizontal postop mid (mm)	Horizontal preop distal (mm)	Horizontal postop distal (mm)
1	1,936	2,631	2,199	2,615	2,738	2,403
2	3,749	3,419	2,033	1,865	2,309	2,942
3	1,704	2,349	1,551	3,339	2,048	2,824
4	1,801	2,453	1,765	2,245	2,112	2,668
5	1,777	2,375	1,192	1,86	1,903	2,015
6	2,31	2,615	1,891	2,013	2,229	2,885
7	1,445	1,928	0,999	1,198	1,517	1,27
8	2,363	2,027	2,14	1,448	2,362	1,883
Mean ± Standard deviation	2.14 ± 0.72	2.47 ± 0.46	1.72 ± 0.44	2.07 ± 0.67	2.15 ± 0.36	2.36 ± 0.59

### Registration of baseline- and follow-up data

Following digital data processing, baseline and 6-month follow-up data were spatially registered (both CBCT scans and IOS). Utilizing an intensity-based medical image registration algorithm (Elastix) baseline and follow-up CBCT scans were aligned with a linear transformation. The same transformation algorithm was applied to register the IOSs.

### Outcome measures

#### Vertical- and horizontal supracrestal soft tissue changes

The primary outcome of the study was to evaluate the vertical supracrestal soft tissue changes at three measurement planes. Coronal, sagittal and axial planes of CBCT scans were oriented in a manner that the sagittal plane became parallel and the coronal plane became perpendicular to the alveolar ridge. In the coronal view window, three planes were selected for measurements (mesial plane: at the line of the most mesial titanium pin used for GBR membrane fixation, distal plane: at the line of the most distal titanium pin, middle plane: halfway between the mesial and distal planes). Supracrestal soft tissue height was measured between the most coronal point of the alveolar crest and the most coronal point of the keratinized alveolar mucosa on both the baseline and the follow-up data.

**Fig. 3:**
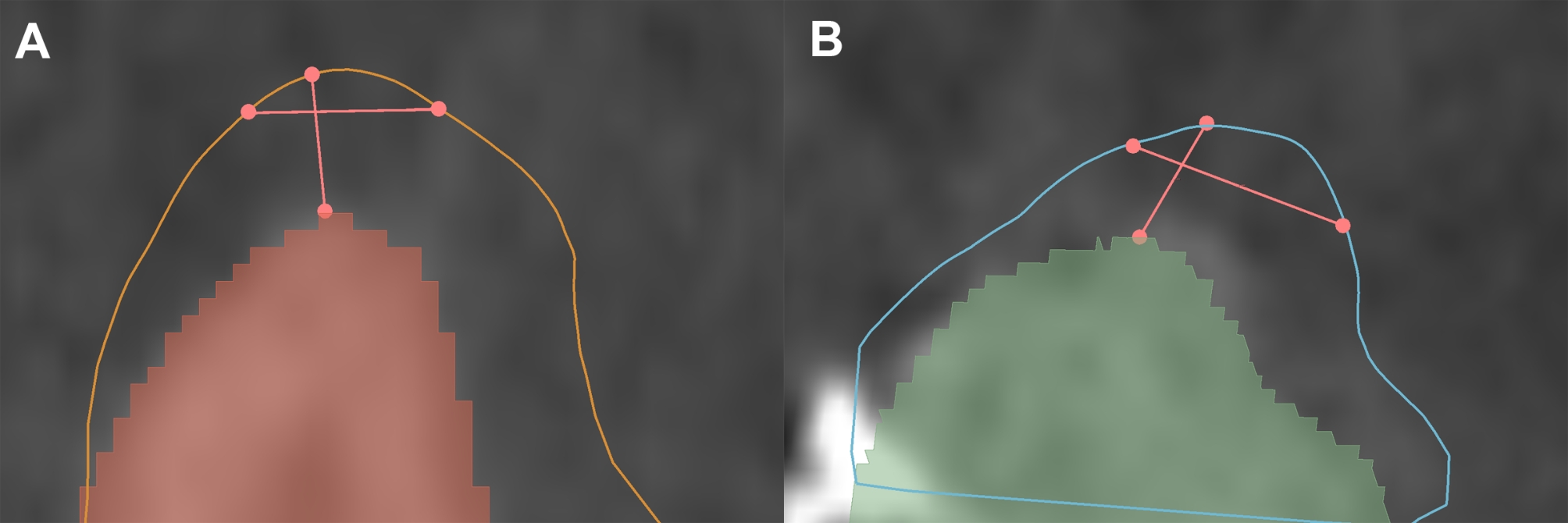
Vertical and horizontal measurements of the supracrestal soft tissue dimensions. (**A**) Baseline (middle plane). (**B**) 6-months follow-up (middle plane)

Additionally to vertical soft tissue dimensions, the bucco-lingual width of the supracrestal soft tissues was measured. Linear measurements were made perpendicular to the long axis of the alveolar crest at the level of the buccal MGJ (visible on IOS) (Fig. 3).

**Table 3:** Horizontal and vertical shifts of the most coronal point of the keratinized mucosa. In the horizontal dimension, positive values indicate that the most coronal point shifted buccaly. In the vertical dimension, positive values indicate that the most coronal point shifted coronally

Patient	Horizontal shift 1 (mm)	Horizontal shift 2 (mm)	Horizontal shift 3 (mm)	Vertical shift 1 (mm)	Vertical shift 2 (mm)	Vertical shift 3 (mm)
1	1,753	1,469	0,901	1,444	2,021	1,407
2	1,323	2,834	2,017	0,332	0,974	1,73
3	2,898	4,001	2,528	-1,355	-0,725	0,156
4	1,771	2,578	2,311	-0,897	-0,587	-0,342
5	1,959	2,325	2,577	-1,355	-1,804	-0,872
6	1,769	2,011	2,664	0,31	0,769	1,011
7	0,736	0,875	1,43	-0,042	-0,296	-0,09
8	0,644	1,111	2,148	-2,102	-1,824	-0,813
Mean ± Standard deviation	1.61 ± 0.72	2.15 ± 1.02	2.07 ± 0.62	-0.45 ± 1.16	-0.18 ± 1.35	0.27 ± 0.99

#### Horizontal and vertical shift of the supracrestal soft tissues

**Fig. 4:**
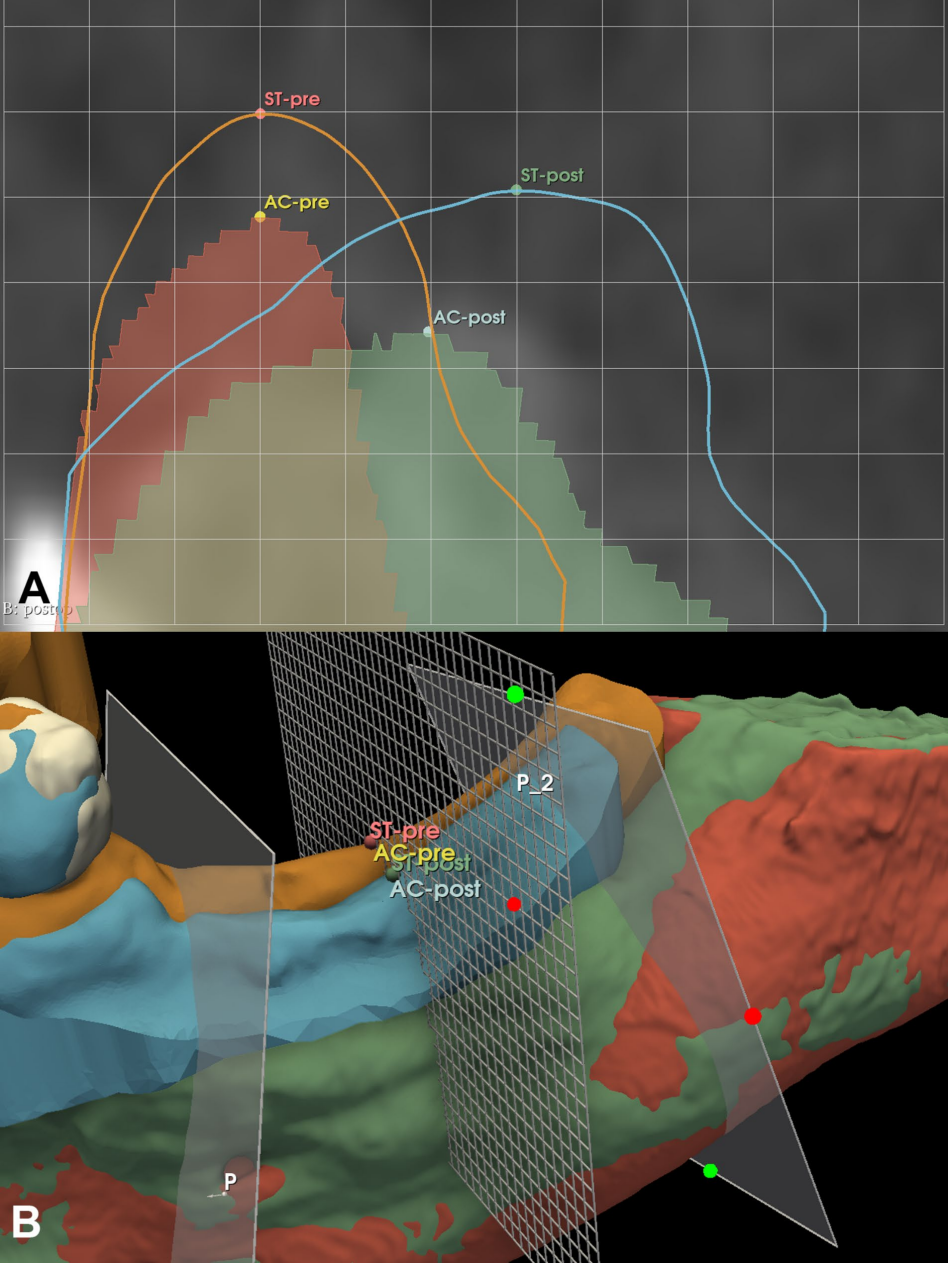
Shift of the keratinized tissue crest (KTC) following hard tissue augmentation. (**A**) Planar view of the measurements (middle plane). (**B**) 3D view of overall soft tissue alterations

A measuring grid with a 1 mm interval was overlayed on the previously mentioned measurement planes (mesial, middle, distal). Two reference points were placed midcrestally at the most coronal level of the keratinized tissue crest (KTC) both on the baseline and 6-month follow-up models (ST-pre; ST-post). Two additional reference points were placed at the top of baseline and follow-up edentulous alveolar crests (AC-pre; AC-post). Horizontal distances between the ST-pre; ST-post points and the corresponding AC reference point were measured to assess the occasional shift of KTC following horizontal GBR (Fig. 4).

### Statistical analysis

Descriptive statistics were used to describe the variables, data were expressed as means ± standard deviations. Statistical differences between baseline and 6-month follow-up supracrestal soft tissue dimensions were analyzed using inferential statistics with a significance level of α = 0.05. Normality of the previously mentioned variables was checked with the Shapiro-Wilk test. Levene’s test was used to check the homogeneity of the variances. Data were found to be normally distributed, and the homogeneity assumption of the variances were met. The continuous variables between subgroups were compared with parametric statistics. The paired sample t-test was utilized to evaluate statistical differences for each variable at different time points. The statistical analysis was performed using the STATA 18 software package (StataCorp LLC, College Station, TX, USA).

## Results

### Patient demographics

Seven systemically healthy patients (6 female, 1 male, aged between 40 and 75 years; mean age 54,7 years) with 8 surgical sites were included in the current case series. Every augmentation procedure was performed in the premolar-molar region of the mandible. Five of the included defects were classified as HL (horizontal-large) and three of the included defects were classified as HM (horizontal- medium).

A slight bucco-lingual discrepancy could be detected between the top of the alveolar crest and the midline of the KTC. In the cross sectional planes the midline of the KTC was located buccally from the midline of the alveolar crest.

### Primary outcome – changes in supracrestal soft tissue height

Baseline supracrestal soft tissue height was measured midcrestally at three measurement planes (Table 1). In the mesial plane baseline supracrestal soft tissue height averaged at 2.37 mm ± 0.68 mm. At the mesial measurement plane vertical soft tissue dimensions showed no statistically significant difference (*p* = 0.21) at 6-month follow-up, being 2.62 mm ± 0.72 mm on average. In the middle plane supracrestal soft tissue height changed from 2.37 mm ± 0.71 mm at baseline to 2.67 mm ± 0.67 mm at 6-month follow-up, however this difference was statistically not significant (*p* = 0.21). Contrary in the distal measurement plane a statistically significant difference (*p* = 0.0002) was detected between the baseline and follow-up vertical soft tissue values, being 2.64 mm ± 0.87 mm and 3.69 mm ± 1.02 mm respectively.

### Secondary outcome measures

The soft tissue width was assessed at the same three measurement planes at the level of the MGJ (Table 2). At the mesial plane supracrestal soft tissue width was measured to be an average of 2.14 mm ± 0.72 mm at baseline compared to 2.47 mm ± 0.46 mm at follow-up. This difference was not found to be statistically significant (*p* = 0.06). Also at the mesial plane, horizontal supracrestal soft tissue dimensions showed no statistically significant difference (*p* = 0.21) between baseline and 6-month follow-up, being 1.72 mm ± 0.44 mm and 2.07 mm ± 0.67 mm respectively. At the distal plane supracrestal soft tissue width change from 2.15 mm ± 0.36 mm at baseline to 2.36 mm ± 0.59 mm at follow-up, which difference was stistically not significant (*p* = 0.28).

The midline of the KTC showed a horizontal substantial shift in the buccal direction (Table 3). The horizontal distance between ST-pre and ST-post showed an average of 1.61 mm ± 0.72 mm, 2.15 mm ± 1.02 mm and 2.07 mm ± 0.62 mm at the mesial, middle and distal planes. Simultaneously, the vertical distance between ST-pre and ST-post showed an average of -0.45 mm ± 1.16 mm, -0.18 mm ± 1.35 mm and 0.27 mm ± 0.99 mm at the mesial, middle and distal planes.

## Discussion

In the current case report, soft tissue alterations following horizontal GBR were investigated utilizing digital models acquired with the combination of segmented CBCT models and IOSs. In the literature the information regarding soft tissue alterations following GBR is scarce, even though soft tissue dimensions at future implant sites contribute substantially to long-term implant success [14, 18]. 

Compared to previous articles [9, 21], our investigation aimed to measure the dimensions of the supracrestal soft tissues before and after hard tissue augmentation and to observe the effects of split thickness flap mobilization during GBR on soft tissue dimensional changes. Measurements taken at three different measurement planes showed approximately 0.3 mm increase of both supracrestal soft tissue height and width, however none of the differences were found to be statistically significant. Except, the vertical soft tissue increase at the distal aspect of the surgical sites were found to be statistically significant, which may be due to the distortion of the retromolar trigone and the mandibular tuberosity following the repositioning of the buccal flap. These findings contradict those previous clinical observations that keratinized soft tissue dimensions are inevitably reduced following augmentation procedures.

In a previous study of our group a slight crestal/ lingual hard tissue resorption following GBR was detected [16]. Another aspect that can influence the vertical increase of keratinized tissues is the applied suturing technique. Due to the double layer suturing, supracrestal keratinized tissues were repositioned coronally.

The observation of a horizontal increase is well in line with the horizontal increase of the underlying alveolar ridge, however compared to previous articles [9, 21] the extent of the horizontal increase is substantially less. This occurrence is more likely, due to the fact that horizontal measurement in our investigation were made in a more coronal level.

Additionally, the buccal horizontal shift of supracrestal keratinized tissues was observed in the current study. It can be emphasized that this horizontal shift may be caused by the buccal displacement of the lingual flap due to the flap mobilization and the horizontal increase of hard tissues as a result of GBR. This buccal shift results in the discrepancy of midlines of the alveolar ridge and the supracrestal soft tissues, requiring a soft tissue shift or occasionally augmentation of the keratinized mucosa during implant uncovery to avoid potentially unfavorable peri-implant soft tissue conditions.

Although our observations on soft tissue dimensional changes following horizontal GBR are unique in the literature, the current study has some drawbacks that have to be addressed. The greatest limitation of the study is the low sample size, therefore, soft tissue changes following horizontal GBR must be investigated on a much larger scale in the future. Another limitation of the current approach is the relatively high possibility of human error during the landmark-based registration of IOSs and CBCT models. In the future this hinderance can be overcome with the automation of the registration process [8]. 

## Conclusions

The current study did not report significant supracrestal soft tissue reduction following horizontal GBR with a split-thickness flap. Even though there was a slight increase in both vertical and horizontal dimensions, differences are clinically negligible. Additionally, the buccal horizontal shift of supracrestal keratinized tissues was observed, which might be caused by the buccal displacement of the lingual flap due to flap mobilization and the horizontal increase of hard tissues. To derive further conclusions on soft tissue changes following ridge augmentation, a study on a larger population has to be conducted.

## Data Availability

No datasets were generated or analysed during the current study.

## Abbreviations

GBR: Guided bone regeneration
MGJ: Mucogingival junction
PIKM: Peri-implant keratinized mucosa
CBCT: Cone-beam computed tomography
IOS: Intraoral scan
e-PTFE: Expanded polytetrafluoroethylene
KTC: Keratinized tissue crest
ST-pre: Preoperative intraoral scan
ST-post: Postoperative intraoral scan
